# Detailed hazard assessment of ethylbenzene to establish an indoor air quality guideline in Japan

**DOI:** 10.1265/ehpm.24-00415

**Published:** 2025-05-09

**Authors:** Kaoru Inoue, Yoko Hirabayashi, Kenichi Azuma

**Affiliations:** 1 Division of Risk Assessment, National Institute of Health Sciences, 3-25-26 Tonomachi, Kawasaki-ku, Kawasaki, Kanagawa 210-9501, Japan; 2 Center for Biological Safety and Research, National Institute of Health Sciences, 3-25-26 Tonomachi, Kawasaki-ku, Kawasaki, Kanagawa 210-9501, Japan; 3 Department of Preventive Medicine and Behavioral Sciences, Faculty of Medicine, Kindai University, 377-2 Ohnohigashi, Sayama, Osaka 589-8511, Japan

**Keywords:** Indoor air quality, Ethylbenzene, Hazard assessment value

## Abstract

**Background:**

Indoor air quality (IAQ) is an important determinant of human health. In Japan, IAQ guidelines have been established for 13 chemicals since 1997. Regarding ethylbenzene (EB), a previous guideline value of 3800 µg/m^3^ was established in 2000. However, the Ministry of Health, Labour, and Welfare decided to revise the value because of the publication of new hazard information after the establishment of the previous guideline value and the establishment of their respective IAQ guidelines by foreign organizations based on the new hazard information. This study conducted a detailed hazard assessment on EB and derived hazard assessment values to provide a toxicologically valid basis for revising the IAQ guideline value.

**Methods:**

As it was defined that the IAQ guidelines would not exert adverse health effects on humans even if they inhaled the chemicals from indoor air over a lifetime, we investigated the general toxicity, developmental and reproductive toxicity, genotoxicity, and carcinogenicity of EB based on reliable hazard information cited in published assessment documents by domestic, foreign, or international risk assessment organizations. All the collected hazard information was examined, and we originally judged the no-observed adverse effect level and the lowest observed adverse effect level of each toxicity study. We then selected the most appropriate key study, an endpoint, and a point of departure and derived the hazard assessment values for each toxicity category. Finally, we selected a representative hazard assessment value for EB from the minimum hazard assessment value among general toxicity, developmental and reproductive toxicity, and carcinogenicity.

**Results:**

Among the three toxicity categories, the minimum hazard assessment value was obtained from general toxicity, which was 0.0858 ppm (370 µg/m^3^) based on the loss of the outer hair cells in the organ of Corti in the cochlea observed in a 13-week repeated-dose inhalation toxicity study using rats.

**Conclusions:**

It would be appropriate to adopt 0.0858 ppm (370 µg/m^3^) as a representative hazard assessment value to provide a basis for revising the IAQ guideline value for EB.

## Graphical Abstract

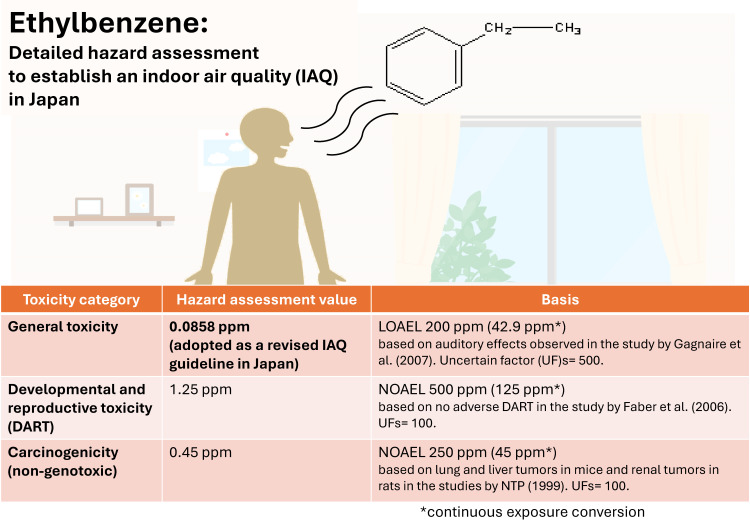

## Background

Indoor air contains microorganisms such as mold, chemical substances and house dust which can cause human health hazard depending on their exposure levels [1–3]. In particular, indoor air quality (IAQ), which is determined by chemical substances released from building materials, is a vital determinant of human health. To protect IAQ, the World Health Organization (WHO), the German Committee on Indoor Air Guide Values in Umweltbundesamt, the French Agency for Food, Environmental, and Occupational Health & Safety (ANSES), and Health Canada each have established their own IAQ guidelines for chemicals that cause indoor air pollution. In Japan, IAQ guidelines have been established for 13 chemicals by the Ministry of Health, Labour, and Welfare (MHLW) since 1997 considering scientific discussions at the Committee on Indoor Air Pollution (CIAP). Azuma et al. reported an outline of the status of efforts in Japan regarding IAQ guidelines [4].

For the future establishment of new or revised IAQ guidelines, the MHLW and CIAP discussed the definitions of the IAQ guidelines once again in 2023 and confirmed that the IAQ guidelines should be those that would not exert adverse health effects on humans even if they inhaled the chemicals from indoor air over a lifetime. The MHLW and CIAP also decided that the IAQ guidelines should be established based on currently available scientific knowledge on toxicities induced by long-term exposure (general toxicity, developmental and reproductive toxicity, and carcinogenicity). Finally, the MHLW and CIAP clarified the concept of the hazard assessment on human health in a detailed risk assessment (the concept of detailed risk assessment for the IAQs) for setting IAQ guidelines to establish a scientifically robust assessment system in January 2024 [5].

Ethylbenzene (EB; CAS No. 100-41-4) is used as a building material, including adhesive, paint, ink, wax, and solvent for waterproofing agents [6]. It is a colorless liquid with an aromatic odor and the following chemical and physical properties: boiling point 136.1 °C, melting point −94.9 °C, density 0.8670 g/cm^3^ at 20 °C, slightly soluble in water (152 mg/L at 20 °C) and chloroform, miscible with diethyl ether and ethanol, volatility at vapor pressure 1.28 kPa at 25 °C, relative vapor density (air = 1) 3.7, and flash-point (closed-cup) at 15 °C [7]. Due to its high vapor pressure and volatility, EB is emitted from building materials into the indoor air as a gaseous substance, resulting in increasing the indoor concentration. Therefore, the major exposure route from building materials to residents is inhalation.

A survey of volatile organic compounds in indoor air conducted in Japan reported the frequent detection of EB [8–10]. In the survey conducted in 2005, indoor and outdoor air samples were collected from 50 different residences from January to February, and their VOC constituents were analyzed using the TD-GC/MS method. In this survey, EB was detected in 49 of 50 residences, and the mean, median, and maximum indoor air concentrations were 5.3, 3.3, and 26 µg/m^3^, respectively [8]. In the survey conducted in the winter of 2012, 2013, and 2014 and in the summer of 2012 and 2013, indoor air samples were collected from 602 houses in Japan [9]. The mean, median, and maximum indoor air concentrations of EB were found to be 5.6, 2.2, and 710 µg/m^3^ in winter and 4.4, 2.3, and 240 µg/m^3^ in summer, respectively. Jung et al. reported another survey in the homes of 5017 randomly selected participants in the Japan Environment and Children’s Study (JECS), a nationwide, prospective birth cohort study that registered 103,099 pregnancies at an early stage through 15 Regional Centers in Japan during 2011–2014 [10–12]. In this survey, 12 VOCs and inorganic gaseous pollutants were measured over 7 days using passive samplers when the children were aged 1.5 and 3 years. The median, 95th percentile, and maximum indoor air concentrations were 3.7, 19, and 570 µg/m^3^ in the 1.5-year survey and 3.5, 18, and 210 µg/m^3^ in the 3-year survey, respectively [10].

According to Azuma et al. [4], the guideline value for EB established in 2000 was 3800 µg/m^3^, which was derived from the no-observed adverse effect level (NOAEL) of 2150 mg/m^3^ based on the effects on the liver and kidneys observed in a 13- to 14-week repeated-dose inhalation toxicity study using rats and mice [13]. After establishing the guideline value for EB in Japan, new hazard information such as a 13-week repeated-dose toxicity study in rats [14] became available, and some foreign organizations established their IAQ guidelines for EB based on the hazard information. For instance, the European Collaborative Action [15], Umweltbundesamt [16], and ANSES [17] established their IAQ guidelines for EB at 0.197 ppm (0.85 mg/m^3^), 0.2 mg/m^3^ and 0.3 ppm (1.5 mg/m^3^), respectively, based on ototoxicity observed in the 13-week repeated-dose toxicity study in rats [14]. Health Canada [18] established the guideline value at 0.4 ppm (2 mg/m^3^) based on various effects on the liver and pituitary gland of mice in a 103-week repeated-dose carcinogenicity study [19]. Based on these information, the MHLW decided to revise the IAQ guideline value for EB.

To provide a basis for revising the IAQ guideline value, the present study conducted a detailed hazard assessment on EB and derived the hazard assessment values according to “The Concept of Detailed Risk Assessment for the IAQs” [5]. The hazard assessment values are similar to the Reference Dose (RfD) or the Reference Concentration (RfC) for US EPA, the Minimum Risk Levels (MRLs) for ATSDR and the Acceptable Daily Intake (ADI) or the Tolerable Daily Intake (TDI) for the FAO/WHO Joint Meeting on Pesticide Residues (JMPR) and on Food Additives (JECFA). The values cover all categories of toxicity, including carcinogenicity.

## Methods

### 1. Collection of hazard information

Essentially, we collected reliable hazard information on EB (e.g., epidemiological studies and toxicity studies using animals) from assessment documents published by domestic, foreign, or international risk assessment organizations. Resource of the reliable hazard information was selected based on a document “Regarding reliability evaluation of toxicity data on human health effects under the Chemical Substances Control Law” [20]. The hazard assessment documents practically collected for the present assessment are shown in Reference [7, 15–18, 21–33]. We also collected published reports on toxicity studies that served as the basis for the hazard assessment values, including the IAQ guidelines derived by the risk assessment organizations. Furthermore, we searched for the latest hazard information on EB and obtained it if available. Besides the hazard information, we investigated and summarized the toxicokinetics of EB.

### 2. Detailed hazard assessment

The collected hazard information on the inhalation exposure route was organized according to toxicity category (general toxicity, developmental and reproductive toxicity, genotoxicity, and carcinogenicity), and the contents of the collected information were carefully examined, evaluated and weighted to select those that would contribute to a quantitative evaluation (deriving toxicity assessment values). We also determined whether the findings observed in each toxicity study were adverse effects, and the NOAEL and the lowest observed adverse effect level (LOAEL) for each study were originally judged. Moreover, we selected the most appropriate key study, endpoint, and point of departure (POD) and derived the hazard assessment values for each toxicity category. During the calculation of the hazard assessment values, continuous exposure conversion of NOAELs/LOAELs was performed to obtain a value equivalent to an exposure level of 24 h and 365 days. For the toxicity with threshold, the hazard assessment values were calculated by dividing the NOAEL/LOAEL by uncertainty factors. For the toxicity without threshold (carcinogenicity for mutagenic substances), the hazard assessment value calculated was 10^−5^ carcinogenic risk level. We finally selected a representative hazard assessment value for EB from the minimum hazard assessment value among the three toxicity categories. These concept and methods were based on “The Concept of Detailed Risk Assessment for the IAQs.” [5]

## Toxicokinetics

EB is readily absorbed after inhalation and oral exposure [34–38], distributed throughout the body, and excreted primarily through urine. Liquid EB is also rapidly absorbed through the skin if volatilization is prevented; however, dermal absorption of vapor appears to be minimal [39–41]. The amount of EB absorbed in humans correlates with the amount of body fat [36, 37, 41, 42]. EB has two distinct metabolic pathways; one is alpha-oxidation by various cytochrome P450 isoenzymes of the side chain and the other is omega-oxidation. In alpha-oxidation, the primary metabolic pathway, 1-phenylethanol (primarily the R-isomer), is produced [43]. Various studies have demonstrated the involvement of hepatic microsomal enzymes in the hydroxylation of EB [43–45], and in microsomes prepared from human liver, the hydroxylation of EB to 1-phenylethanol is catalyzed by the cytochrome P-450 isoforms CYP2E1 and CYP2B6 [45]. EB is excreted in urine primarily in the form of water-soluble oxidation products. The excretion pattern of urinary metabolites varies among mammalian species. For instance, in humans, EB is excreted in urine as mandelic acid and phenylglyoxylic acid, whereas in rats and rabbits, it is primarily excreted as hippuric acid and phenaceturic acid [35, 36, 39, 42, 46, 47].

## Effects

### 1. General toxicity

#### 1) In humans

Although there are some reports on the systemic effects of EB in humans, details such as exposure period and concentration could not be confirmed, and some of the reports described combined exposure with other substances or noise [48, 49]. Therefore, these epidemiological studies were judged invalid for the quantitative evaluation of general toxicity in humans. These studies have confirmed respiratory and eye irritation, ototoxicity (hearing loss), and hematological changes (increased lymphocyte count and decreased hemoglobin concentration) in humans. In one of the epidemiological studies reporting an auditory effect caused by EB and noise [48], a survey of workers in two petrochemical plants in China revealed that 78.4% of 246 workers exposed to EB by inhalation at 122.83 ± 22.86 mg/m^3^ (28.3 ± 5.1 ppm) and a mean noise of 82.7 dB (A) (cumulative noise exposure over 20 years) had a hearing loss of ≥25 dB. Moreover, 80.1% of 307 workers exposed to EB by inhalation at 134.64 ± 31.97 mg/m^3^ (31.0 ± 7.4 ppm) and a mean noise of 83.5 dB (A) had a hearing loss of ≥25 dB. Benzene, toluene, styrene, and xylene concentrations in plants were found to be below the detection limit (<0.2–0.8 ppm). As a control group, 5.2% of 327 office workers exposed to an average noise level of 67.3 dB (A) had hearing loss, and 56.9% of 290 power plant workers exposed to an average noise level of 84.3 dB (A) had hearing loss. The odds ratios of hearing loss (25 dB or more) for office workers in the control group were 86.4 (95% CI: 28.4–452) and 124 (95% CI: 11.7–651), which were significantly higher than those in the control group. Furthermore, there were significant differences in various neurobehavioral function tests (digital span and simple reaction time) compared with the control group [48].

#### 2) In animals

There were eight studies of repeated-dose inhalation toxicity of EB (Table 1). Our judgement of NOAELs/LOAELs of each study based on the examination of the study results is also presented in Table 1.

**Table 1:** Summary of repeated-dose toxicity studies on EB and our determined NOAELs/LOAELs

**Animal**	**Exposure**	**Dose**	**Endpoint**	**NOAEL/LOAEL (Continuous exposure conversion)**	**Reference**
SD rats (Males only, 14 animals/group)	13 weeks (6 h/day, 6 days/week)	200, 400, 600, and 800 ppm	Loss of the third row of the outer hair cells (OHCs) in the organ of Corti in the cochlea	LOAEL 200 ppm (42.9 ppm)	Gagnaire et al., 2007. [14]
Wag/Rij rats (Males only)	One or 13 weeks (8 h/day, 5 days/week)	300–800 ppm	Dose-dependent loss of OHCs	Not determined	Cappaert et al., 2001. [50]
Fischer 344 rats (Males and females, 10 animals/sex/group) (equivalent to OECD TG 413, GLP study)	13–14 weeks (6 h/day, 5 days/week)	100, 250, 500, 750, and 1000 ppm	No adverse effects	NOAEL 1000 ppm (178.6 ppm)	NTP, 1992. [13]
B6C3F1 mice (Males and females, 10 animals/sex/group) (equivalent to OECD TG 413, GLP study)	13–14 weeks (6 h/day, 5 days/week)	100, 250, 500, 750, and 1000 ppm	No adverse effects	NOAEL 1000 ppm (178.6 ppm)	NTP, 1992. [13]
Wistar rats (Males and females, 18 animals/sex/group)	12 weeks (6 h/day, 5 days/week)	100 ppm	No adverse effects	NOAEL 100 ppm (17.9 ppm)	Clark, 1983. [51]
Fischer 344 rats (Males and females, 50 animals/sex/group) (equivalent to OECD TG 451, GLP study)	104 weeks (6 h/day, 5 days/week)	75, 250, and 750 ppm	Significant increase in the severity of chronic progressive nephropathy in females	NOAEL 75 ppm (13 ppm)	NTP, 1999. [19]
B6C3F1 mice (Males and females, 50 animals/sex/group) (equivalent to OECD TG 451, GLP study)	103 weeks (6 h/day, 5 days/week)	75, 250, and 750 ppm	Syncytial alteration of hepatocytes in males and hyperplasia of the pituitary gland pars distalis in females	NOAEL 75 ppm (13 ppm)	NTP, 1999. [19]
B6C3F1mice F344 rats New Zealand White rabbits (Males and females, 5 animals/sex/group)	4 weeks (6 h/day, 5 days/week)	Rats and mice: 99, 382, and 782 ppm Rabbits: 382, 782, and 1610 ppm	No adverse effects	Rats and mice: NOAEL 782 ppm (139.6 ppm) Rabbits: NOAEL 1610 ppm (287.5 ppm)	Cragg et al., 1989. [52]

In the 13-week repeated-dose study by Gagnaire et al. [14], auditory effects such as auditory thresholds and number of hair cells in the organ of Corti in the cochlea were measured in male rats that inhaled EB at a concentration of up to 800 ppm. The most susceptible toxicity endpoint was the loss of outer hair cells (OHCs) since this finding was detected at a lower concentration than the LOAEL for the change in auditory threshold. In the 200-ppm group, loss of the third row of OHCs (up to 30%) was observed in four of eight animals, and the loss of almost all OHCs was also observed in the 600- and 800-ppm groups. Loss of OHCs is a general cause of auditory disorders. Because the dose-dependent loss of OHCs (25%–66%) was also detected in the 13-week study by Cappaert et al. [50], we considered that this finding was a toxicologically significant effect of EB, although the auditory disorder and OHC alterations were not investigated in the standard 13-week study conducted by the NTP [13]. Therefore, we judged that 200 ppm (continuous exposure conversion: 200 × 6/24 × 6/7 = 42.9 ppm) was the LOAEL of that study [14].

In the 13-week repeated-dose studies using rats and mice conducted by the NTP [13], all required tests such as hematological and serum biochemical examinations and histopathological examinations were performed, which revealed an increase in the absolute and/or relative weights of the liver or kidneys. However, no toxicologically significant changes were detected in the serum biochemical and histopathological examination related to the liver and kidneys, and the kidney weights in rats also showed no dose-dependency. Therefore, we considered that the increase in the liver and kidney weights was not toxicologically significant and hence judged that 1000 ppm (continuous exposure conversion: 1000 × 6/24 × 5/7 = 178.6 ppm) was the NOAEL of the studies conducted by the NTP [13].

The 12-week repeated-dose study by Clark [51] examined the clinical symptoms, body weight and food intake, hematological and serum biochemical parameters, urinalysis, organ weights, and histopathology in the major organs and found no statistically significant changes related to the treatment in any test items. Therefore, we judged that 100 ppm was the NOAEL of the study by Clark [51]. Nevertheless, because this study was conducted with a single dose, we considered it unsuitable for dose–response analysis.

In the 104-week repeated-dose study in rats conducted by the NTP [19], chronic progressive nephropathy was detected in male and female rats of all groups, including the controls, although its incidence showed no statistically significant difference between the control and treatment groups in both sexes. However, the severity of chronic progressive nephropathy showed a statistically significant increase in female rats from the lowest dose of 75 ppm, as reported by the NTP [19]. Since the statistical analysis method for the severity of chronic progressive nephropathy in this study was for a two-group comparison, we obtained the original data on the severity of chronic progressive nephropathy and conducted a statistical analysis for multiple comparisons (Steel–Dwass test) using the KyPlot 6.0 software. Our results confirmed a statistically significant difference in the severity of the lesion from concentrations of ≥250 ppm. Therefore, we judged that 75 ppm (continuous exposure conversion: 75 × 6/24 × 5/7 = 13.39 ppm ≒ 13 ppm) was the NOAEL of general toxicity in this study based on the severity of chronic progressive nephropathy in female rats.

Furthermore, the 104-week repeated-dose study in mice conducted by the NTP [19] showed a statistically significantly increased incidence of syncytial alteration (multinucleation) of hepatocytes and hyperplasia in the anterior pituitary gland in males and females, respectively, from concentrations of ≥250 ppm. At 750 ppm, there were statistically significantly increased incidences of hyperplasia of thyroid follicular cells in both sexes, centrilobular hepatocellular hypertrophy, hepatocellular necrosis, and alveolar epithelial metaplasia in males and eosinophilic foci of altered hepatocytes in females. Hence, we judged that 75 ppm (continuous exposure conversion: 75 × 6/24 × 5/7 = 13.39 ppm ≒ 13 ppm) was the NOAEL of general toxicity in this study based on the findings observed at ≥250 ppm.

Cragg et al. [52] conducted 4-week repeated-dose studies in rats, mice, and rabbits and observed the sporadic occurrence of salivation and tearing in rats, increase in the absolute and/or relative liver weights of male and female rats and female mice, and increase in platelet count or total number of white blood cells in male and female mice at the mid-dose (382 ppm) or the highest dose (782 ppm). Regarding the liver, no histopathological findings were detected in rats and mice. In rabbits, no toxicological findings were detected in the weights and histopathology of the liver. Therefore, we considered that the findings observed in each animal of this study were not adverse effects and that the highest doses (782 ppm in rats and mice and 1610 ppm in rabbits) in each animal were the NOAELs of this study. After the continuous exposure conversion, the NOAELs were 139.6 ppm (= 782 × 6/24 × 5/7) for rats and mice and 287.5 ppm (1610 × 6/24 × 5/7) for rabbits. Nevertheless, the results of this study were inappropriate to adopt as a base of hazard assessment value due to the short treatment period.

Elovaara et al. [53] also conducted a 16-week repeated-dose study in rats. However, because the purpose of their study was to examine metabolic enzyme levels in the liver and kidneys, we did not evaluate their study results for the present quantitative assessment.

### 2. Developmental and reproductive toxicity (DART)

#### 1) In humans

We found no significant epidemiological information on DART induced by EB exposure via inhalation.

#### 2) In animals

We found eight reports on DART studies for EB (Table 2). Our judgement of NOAELs/LOAELs of each study based on the examination of the study results is also presented in Table 2.

**Table 2:** Summary of DART studies on EB and our determined NOAELs/LOAELs

**Study type and Animal**	**Exposure**	**Dose**	**Endpoint**	**NOAEL/LOAEL (Continuous exposure conversion)**	**Reference**
Two-generation reproductive toxicity study (OECD TG 416, GLP study) SD rats (Males and females, 30 animals/sex/group: P/F1 generation)	Males: 70 days before mating Females: 70 days prior to, through gestation day 20 and from lactation day 5–21. (6–7 hrs/day, 7 days/week)	25, 100, and 500 ppm (For females on lactation days 1–4, gavaged at 26, 90 and 342 mg/kg bw/day)	No adverse effects observed.	NOAEL 500 ppm (125 ppm) for parents and offsprings	Faber et al., 2006. [54]
Repeated dose toxicity study Fischer 344 Rats and B6C3F1 mice (equivalent to OECD TG 413, GLP study)	13 weeks (6 h/day, 5 days/week)	100, 500, and 1000 ppm	No adverse effects on sperm, testicular morphology, length of estrus cycle, spermatid counts, sperm motility, caudal or epididymal weights.	Not determined	NTP, 1992. [13]
Developmental toxicity study Wistar rats (78–107 animals/group)	Gestation days (GD) 1–19 or 3 weeks before mating and whole pregnant period (7 hrs/day, 7 days/week)	100 and 1000 ppm	Dams: No adverse effects Fetus: increased incidence of skeletal variations (supernumerary ribs).	Dams: NOAEL 1000 ppm (292 ppm) Fetuses: NOAEL 100 ppm (29 ppm)	Andrew et al., 1981; Hardin et al., 1981. [55, 56]
Developmental toxicity study New Zealand white rabbits (29 or 30 animals/group)	GD 1–24 (7 hrs/day, 7 days/week)	100 and 1000 ppm	No adverse effect	NOAEL 1000 ppm (292 ppm) for Dams and fetuses	Andrew et al., 1981. [55]
Developmental neurotoxicity study SD rats (F2 generation in the two-generation DART study (Faber et al. 2006))	See the two-generation reproduction toxicity study.	See the study by Faber et al., 2006	No adverse effects in functional tests (FOB), motor function tests, acoustic startle response tests, learning and memory tests, and histopathological examinations in the brain and nervous system.	Not determined	Faber et al. 2006. [54], MAK, 2012. [25]
Developmental toxicity study (OECD TG 414) SD rats (21–25 animals/group)	GD 6–20 (6 hrs/day)	100, 500, 1000, and 2000 ppm	Dams: Decrease of body weight and food consumption Fetuses: decrease of body weight and skeletal variations.	NOAEL 500 ppm (125 ppm) for general toxicity in Dams and developmental toxicity in fetuses NOAEL 2000 ppm (500 ppm) for teratogenicity	Saillenfait et al., 2003. [57]
Developmental toxicity study SD rats (15–19 animals/group)	GD 6–20 (6 hrs/day)	250 and 1000 ppm	Dams: decrease of body weight gain Fetuses: No adverse effects	Dams: NOAEL 250 ppm Fetuses: NOAEL 1000 ppm (250 ppm)	Saillenfait et al., 2006. Saillenfait et al., 2007. [58, 59]
Developmental toxicity study* CFY rats CFLP mice New Zealand White rabbits	Rats: GD 7–15 (24 hrs/day) Mice: GD 6–15 (24 hrs/day) Rabbits: GD 7–20 (24 hrs/day)	Rats: 600, 1200, and 2400 mg/m^3^ (138, 277, and 554 ppm) Mice: 500 mg/m^3^ (115 ppm) Rabbits: 500 and 1000 mg/m^3^ (115 and 231 ppm)	Rats: Increase of death and absorbed embryo, delayed ossification, decrease of body weight gain, malformation of urinary tract and skeletal abnormalities Mice: Malformation of urinary tract etc. Rabbits: Decrease of body weight in fetuses, abortion	Not determined	Ungváry and Tátrai, 1985. [60]

*Since details of some study results were not be able to confirmed, this information was treated as a reference one.

In the two-generation reproductive toxicity study by Faber et al. [54], there were no adverse reproductive or developmental toxicity effects in any treatment group. Transient suppression of body weight gain in male parent animals and an increase in liver weight in male and female parent animals of the second generation were detected in the highest-dose group. However, because the liver showed no histopathological changes, we considered that the changes in the liver weight were an adaptive response. Therefore, we judged that 500 ppm (continuous exposure conversion: 500 × 6/24 = 125 ppm) was the NOAEL for both parents and pups in the two-generation reproductive toxicity study.

In the 13-week repeated-dose toxicity study conducted by the NTP [13], no adverse effects on sperm, testicular morphology, length of estrus cycle, spermatid counts, sperm motility, and caudal or epididymal weights were observed in rats and mice treated with EB at concentrations of up to 1000 ppm. Since reproductive and developmental toxicity could not be detected in this study, we did not determine the NOAEL for DART based on this study.

In the developmental toxicity studies using rats conducted by Andrew et al. and Hardin et al. [55, 56], on day 21 of gestation (GD21), no pathological findings were observed in any of the organs of the dams treated with up to 1000 ppm EB during gestation. Dams treated before mating showed no effects on reproductive parameters. The absolute and relative weights of the liver, kidney, and spleen of the dams in the 1000-ppm group were significantly higher (22%, 10%, and 10% increases, respectively), but there were no histopathological changes in these organs. In the fetuses, the incidence of skeletal variations (supernumerary ribs) increased significantly in the 1000-ppm group. In our assessment, we considered that the increase in organ weights of the dams was not toxicologically significant, and the NOAEL for the dams treated during gestation was judged to be 1000 ppm (continuous exposure conversion: 1000 × 7/24 = 292 ppm). We also judged that 100 ppm (continuous exposure conversion: 100 × 7/24 = 29 ppm) was the NOAEL for fetuses based on the statistically significant increase in the incidence of skeletal variations (supernumerary ribs) at 1000 ppm.

In the developmental study using rabbits conducted by Andrew et al. [55], no adverse effects were observed in the treated dams, especially in the histopathological examination. Fetuses of the treatment groups also showed no effects on development. The numbers of implantation, death, or resorption per litter showed no statistically significant difference from those of the control group. Prenatal mortality (5%–8%) and preimplantation loss (18%–27%) showed no dose-dependency. Therefore, we judged that 1000 ppm (continuous exposure conversion: 1000 × 7/24 = 292 ppm) was the NOAEL for both parents and fetuses in this developmental toxicity study in rabbits.

The developmental neurotoxicity study conducted using an F2 generation of the two-generation reproduction toxicity study [54] reported no treatment-related effects in the functional examination (FOB) (on Days 4, 11, 22, 45, and 60 after birth), motor function test (on Days 13, 17, 21, and 61 after birth), auditory startle response test (on Days 20 and 60 after birth), learning and memory test using the Biel water maze (on Days 26 and 62 after birth), and morphometric and histological examination of the brain and nervous system (on Days 21 and 72 after birth).

In the developmental toxicity study conducted using rats [57], there were no deaths, but clinical signs (ataxia and decreased activity) were observed in the dams of the 2000-ppm group. Moreover, dams of the ≥1000 ppm groups exhibited significantly lower body weights, suppressed weight gain, and decreased food intake during the treatment period. No statistically significant differences were observed in the pregnancy rate, number of corpora lutea, and number of implantations between the treatment and control groups. In the 2000-ppm group, there was an increase in the number of dead fetuses and resorption, but without any statistical significance. Although the size of fetuses showed no significant difference between the control and treatment groups, the body weight of fetuses decreased in a dose-dependent manner in the 1000- and 2000-ppm groups. In the same treatment groups, the number of fetuses with skeletal variations increased. In the other test items in the fetuses, such as the number of live fetuses, sex ratio, and external appearance, no toxicological findings were detected. One or a few cases of visceral malformations were detected in the 100-, 1000-, and 2000-ppm groups; however, there was no dose relationship or a statistically significant difference. Teratogenicity was not detected in the fetuses under the study conditions. Therefore, we judged that the NOAEL for general toxicity in the dams and for developmental toxicity in the fetuses was 500 ppm (continuous exposure conversion: 500 × 6/24 = 125 ppm), based on decreased body weight gain and food intake in the dams and decreased body weight and increased incidence of skeletal variations in the fetuses. The NOAEL for teratology was also determined to be 2000 ppm (continuous exposure conversion: 2000 × 6/24 = 500 ppm).

The developmental toxicity studies using rats [58, 59] reported a decrease in body weight gain in the dams of the 1000-ppm group. Fetuses in the same group showed lower body weight; however, there were no treatment-related toxicological effects on the number of implantations, live fetuses, or resorptions. There were also no teratogenic effects and fetal deaths in the treatment groups. Therefore, we judged that the NOAEL for general toxicity in the dams was 250 ppm (continuous exposure conversion: 250 × 6/24 = 62.5 ppm), and the NOAEL for fetuses was 1000 ppm (continuous exposure conversion: 1000 × 6/24 = 250 ppm).

We obtained information on developmental toxicity studies in rats, mice, and rabbits reported by Ungvary and Tatrai [60]. However, because of the difficulty to confirm details on some of the results, these studies were not evaluated in the present assessment.

### 3. Genotoxicity

Regarding mutagenicity *in vitro*, the reported test results obtained using bacteria (*Salmonella typhimurium* and *Escherichia coli*) and yeast cells (*Saccharomyces cerevisiae*) indicated that EB is not mutagenic with or without metabolic activation [61–68]. Some positive results were obtained in the forward mutation assays using mouse lymphoma cells at cytotoxic concentrations [69, 70]; however, in most of the same assays performed at suitable concentrations, the results were negative with or without metabolic activation [70, 71]. Chromosomal aberration tests using the Chinese hamster ovary (CHO) cell lines and rat liver RL1 cells were negative with or without metabolic activation at the noncytotoxic concentrations [13, 19, 61]. In a cell transformation assay using the Syrian hamster embryo (SHE) cell line, a positive result was detected at the cytotoxic concentration after 7-day incubation; however, no significant change was observed after 24-h incubation [72]. In a sister chromatid exchange assay, a marginal positive response was observed at a cytotoxic concentration in human lymphocytes without reproducibility, and negative results were obtained in CHO cells at the noncytotoxic concentration with or without metabolic activation [19, 68].

In *in vivo* assays, micronuclei formation was not detected in the peripheral blood erythrocytes of B6C3F1 mice exposed to 500–1000 ppm EB 6 h/day, 5 days/week, for 13 weeks [13, 19] or in the polychromatic erythrocytes in the bone marrow of NMRI mice administered two daily doses of 0.37–0.75 mL/kg of EB via intraperitoneal injection [73]. Moreover, unscheduled DNA synthesis was not induced in the hepatocytes of mice that inhaled 375–1000 ppm EB for 6 h [74]. We found no information on genotoxicity in humans exposed to EB alone.

### 4. Carcinogenicity

#### 1) Qualitative evaluation

The classification of the carcinogenicity of EB by different organizations is shown in Table 3.

**Table 3:** Classification of the carcinogenicity of EB

**Organization**	**Classification**	**Definition**	**Reference No.**
IARC (2000)	Group 2B	Possibly carcinogenic to humans	7
US EPA IRIS (1988)	Class D	Not classifiable as human carcinogen	30
ACGIH (2011)	Category A3	Confirmed animal carcinogen with unknown relevance to humans	22
JSOH (2001)*^1^	Class 2B	Probably or possibly carcinogenic to humans (agents with less (possible carcinogenicity to humans)	27
DFG MAK (2011)*^2^	Category 4	Substances that cause cancer in humans or animals or that are considered carcinogenic for humans and for which a MAK value*^3^ can be derived. A nongenotoxic mode of action is of prime importance and genotoxic effects play no or at most a minor part provided the MAK and BAT values are observed.	25
GHS Classification by the Japanese government (2021)	Category 2	Suspected human carcinogens	26

*^1^: It was determined that a revision of the classification was not required as a result of reevaluation in 2020.

*^2^: The cancer category was reaffirmed when the MAK value was set in 2011.

*^3^: MAK value is the maximum workplace concentration of a chemical substance in the workplace air that generally does not exert known adverse effects on the health of the employee nor cause unreasonable annoyance (e.g., by a nauseous odor) even when the person is repeatedly exposed during long periods, generally for 8 h daily but assuming on average a 40-h working week.

Abbreviations: IARC, International Agency for Research on Cancer; ACGIH, American Conference of Governmental Industrial Hygienists; DFG, Deutsche Forschungsgemeinschaft (German Research Foundation); GHS, Globally Harmonized System of Classification and Labeling of Chemicals.

#### 2) Quantitative evaluation

##### 2-1) In humans

The study conducted by Bardodej and Cirek [75] reported no case of malignant tumors in workers exposed to EB for 20 years in a manufacturing plant. The estimated exposure was 6.4 mg/m^3^; however, there were no detailed data on the actual exposure levels. Therefore, the conclusion of no correlation between EB exposure and carcinogenicity in humans was not certain. No other information on carcinogenicity in humans by EB exposure was available.

##### 2-2) In animals

The information obtained on carcinogenicity studies using mice and rats is summarized in Table 4.

**Table 4:** Summary of carcinogenicity studies of EB and our determined NOAELs/LOAELs

**Study type and animal**	**Exposure**	**Dose**	**Endpoint**	**NOAEL/LOAEL (continuous exposure conversion)**	**Reference**
Carcinogenicity Study (equivalent to OECD TG 451, GLP study) B6C3F1 mice (Males and females, 50 animals/sex/group)	103 weeks (6 h/day, 5 days/week)	75, 250, and 750 ppm	Males: Increased incidence of bronchioloalveolar adenoma and adenoma/carcinoma Females: Increased incidence of hepatocellular adenoma and adenoma/carcinoma	NOAEL 250 ppm (45 ppm)	NTP, 1999. [19]

Carcinogenicity study (equivalent to OECD TG 451, GLP study) F344/N rats (Males and females, 50 animals/sex/group)	104 weeks (6 h/day, 5 days/week)	75, 250, and 750 ppm	Males: Increased incidence of renal cell adenoma, adenoma/carcinoma, and interstitial cell tumor in the testes Females: Increased incidence of renal cell adenoma	NOAEL 250 ppm (45 ppm)	NTP, 1999. [19]

In the carcinogenicity study in mice conducted by the NTP [19], the incidence of bronchioloalveolar adenoma (16/50 cases, 32%) and the combined incidence of bronchioloalveolar adenoma and carcinoma (19/50 cases, 38%) significantly increased in males in the 750-ppm group compared with that in the control group. Female mice showed no significant increase in the incidence of lung tumors. Furthermore, the incidence of hepatocellular adenoma (16/50 cases, 32%) and the combined incidence of hepatocellular adenoma and carcinoma (25/50 cases, 50%) significantly increased in females of the 750-ppm group compared with that in the control group. In male mice, there was no increase in the incidence of hepatocellular tumors. The incidences of lung tumors in male mice and liver tumors in female mice statistically significantly increased and were close to the upper limit of the background range (bronchioloalveolar adenoma in males: 14.9% ± 7.0%, 6%–36%; bronchioloalveolar adenoma and carcinoma in males: 21.7% ± 8.0%, 10%–42%; hepatocellular adenoma in females: 12.2% ± 9.7%, 0%–40%; hepatocellular adenoma and carcinoma in females: 21.3% ± 11.9%, 3%–54%). However, we could not confirm the details on the derivation of historical control data in the study by NTP [19], and the incidences of each tumor did not exceed the background data range. Considering that the NTP concluded that EB showed some evidence of carcinogenic activity in male and female mice based on the results of this study [19] and that the derivation of the historical control data was unclear, we concluded that the statistically significantly increased incidences of lung or liver tumors in male or female mice were toxicologically significant. Therefore, we judged that the NOAEL for carcinogenicity in this study was 250 ppm (continuous exposure conversion: 250 × 6/24 × 5/7 = 44.6 ≒ 45 ppm).

In the carcinogenicity study in rats [19], renal cell adenoma, renal cell adenoma and/or carcinoma, and interstitial cell tumor in the testes showed increased incidences in male rats of the 750-ppm group. The incidence of interstitial cell tumor in the testes, known as a spontaneous tumor in aged male F344 rats, at 750 ppm (44/50 cases, 88%) slightly exceeded the historical control data (range: 54%–83%). An increased incidence of renal cell adenoma was also observed in female rats at 750 ppm, accompanied by an increased incidence of renal cell hyperplasia. Therefore, we judged that the NOAEL for carcinogenicity in this study was 250 ppm (continuous exposure conversion: 250 × 6/24 × 5/7 = 44.6 ≒ 45 ppm).

## Evaluation

### 1. General toxicity

Among the repeated-dose toxicity studies described previously, the lowest NOAEL was 75 ppm (continuous exposure conversion: 13 ppm) obtained from the 104-week repeated-dose study using rats and mice performed by the NTP [19], which had sufficient exposure periods to evaluate chronic effects. If this NOAEL is selected as the POD, the following hazard assessment value is suggested:

$$\begin{array}{rl} & 13\;\text{ppm}÷\text{uncertain factors (UFs) 100 (interspecies }\\ & \text{variability 10, intraspecies variability 10)}=0.13\;\text{ppm} \end{array}$$

Conversely, adverse auditory effects, which have also been observed in humans, were detected in the studies by Gagnaire et al. [14] and Cappaert et al. [50]. If the LOAEL of 200 ppm (continuous exposure conversion: 42.9 ppm) in the study by Gagnaire et al. [14] is selected as the POD, the following hazard assessment value is suggested:

$$\begin{array}{rl} & 42.9\;\text{ppm}÷\text{UFs 500 (interspecies variability 2.5, }\\ & \text{intraspecies variability 10, short study period 2, }\\ & \text{use of a LOAEL 10)}=0.0858\;\text{ppm} \end{array}$$

Finally, we determined that it would be appropriate to adopt 0.0858 ppm (370 µg/m^3^) as the representative hazard assessment value for general toxicity. This value is smaller and can take into account the auditory effect, which is considered the most significant adverse effect of EB and observed in both humans [48, 49] and animals [14, 50].

In the derivation of the hazard assessment value of 0.0858 ppm for general toxicity, the UF for interspecies variability was set at 2.5. This is the result obtained by dividing the UF for interspecies variability into toxicokinetics 4 and toxicodynamics 2.5 by the “Concept of Detailed Risk Assessment for the IAQs.” For toxicokinetics, the blood–gas partition coefficients for humans and rats are 28 and 30, respectively [76], and the ratio is approximately 1. We considered that there was almost no interspecies variability in absorption from the lungs to the blood or transfer from the blood to the auditory organs. Therefore, we considered it appropriate to set the UF for interspecies variability in toxicokinetics at 1. Conversely, for toxicodynamics, we could not obtain data on interspecies variability between humans and rats. Therefore, we considered it appropriate to adopt the default value of 2.5 as the UF for interspecies variability in toxicodynamics.

Furthermore, the UF for the insufficient exposure period was set at 2. In the study by Gagnaire et al. [14], the morphological finding (loss of OHCs in the organ of Corti) was detected at a lower concentration than the concentration at which adverse functional effects (increased audiometric thresholds detected by the brainstem auditory-evoked responses) were observed. Moreover, the exposure concentration, rather than the duration of exposure, was considered the cause of the adverse auditory effects. Therefore, we considered it appropriate to adopt the UF at 2 for the insufficient exposure period.

Hearing loss was observed in humans exposed simultaneously to approximately 30 ppm (28.3 ± 5.1 ppm) of EB and noise, with the loss being more severe in the case of simultaneous exposure than in the case of noise exposure alone, indicating that EB would promote the hearing loss by noise [48]. If we adopt 30 ppm (continuous exposure conversion: 30 × 8/24 × 5/7 = 7.14 ppm) as the LOAEL for this epidemiological study and apply UF 100 (10 for intraspecies variability, 10 for the use of the LOAEL), the hazard assessment value would be 0.0714 ppm. This assessment value cannot be adopted as an IAQ guideline value due to the simultaneous exposure to noise. Nevertheless, it was considered that the adopted hazard assessment value of 0.0858 ppm (equivalent to 370 µg/m^3^) for general toxicity might prevent adverse auditory effects in humans.

In the final study report by the NTP [19], there was a statistically significant increase in the severity of chronic progressive nephropathy from the lowest EB dose in female rats in the 104-week study. However, because the statistical method used in the final report was for the comparison of two groups, we considered it inappropriate for our assessment. Therefore, we obtained the original data and analyzed them using a multiple comparison method (Steel–Dwass test using the KyPlot 6.0 software). Consequently, we observed a statistically significant difference for this finding ≥250 ppm, and the NOAEL of this study was judged as 75 ppm in the present assessment.

### 2. Developmental and reproductive toxicity (DART)

Among the several study results obtained, the two-generation reproductive toxicity study by Faber et al. [54] was the only one that could evaluate transgenerational effects. Therefore, the NOAEL of this study, 500 ppm (continuous exposure conversion: 125 ppm), was selected as the POD to calculate the hazard assessment value for DART, as follows:

$$\begin{array}{rl} & 125\;\text{ppm}÷\text{UFs 100 (interspecies variability 10, }\\ & \text{intraspecies variability 10)}=1.25\;\text{ppm} \end{array}$$

Among the several developmental toxicity studies, the NOAEL of 100 ppm (continuous exposure conversion: 29 ppm) based on skeletal variations (supernumerary ribs) in the fetuses of the studies conducted by Andrew et al. and Hardin et al. [55, 56] was lower than the NOAEL of 500 ppm in the two-generation reproductive toxicity study conducted by Faber et al. [54], the key study for the hazard assessment value. Andrew et al. and Hardin et al. [55, 56] reported that the common ratio of doses was large and the LOAEL for the supernumerary ribs of fetuses was 1000 ppm. Therefore, a “genuine” NOAEL for this finding on fetuses might be between 100 and 1000 ppm. It was confirmed that the same findings were not observed at 500 ppm, whereas the number of fetuses with skeletal abnormalities increased at ≥1000 ppm in the rat developmental toxicity study conducted by Saillenfait et al. [57]. Considering these findings, the possibility of the induction of skeletal variations in humans could be avoided when the NOAEL of 500 ppm derived from the two-generation reproductive toxicity study by Faber et al. [54] was selected as the POD for a hazard assessment value on DART.

According to the JSOH [27], EB was considered a DART chemical in Group 2 (a chemical considered possibly toxic for human reproduction) because some studies indicated toxicity affecting the development of the next generation [55–57].

### 3. Genotoxicity

As described previously, almost all results in the *in vitro* and *in vivo* assays for EB were negative. Although there were positive results in some *in vitro* studies, the cytotoxicity and the lack of reproducibility might be involved in their positive results, indicating the possibility of false-positive results. In conclusion, we considered that EB is nongenotoxic (nonmutagenic) based on the review of all study results. AU NICNAS and ATSDR also judged that EB is not genotoxic both *in vitro* and *in vivo* [21, 23].

### 4. Carcinogenicity

As described earlier, we judged that EB is nongenotoxic (nonmutagenic). Therefore, it was considered that the carcinogenicity of EB observed in mice and rats has a threshold.

In both carcinogenicity studies in mice and rats [19], since the NOAEL was judged to be 250 ppm (continuous exposure conversion: 250 × 6/24 × 5/7 = 45 ppm), we selected this NOAEL as the POD and suggested the following hazard assessment value:

$$\begin{array}{rl} & 45\;\text{ppm}÷\text{UFs 100 (interspecies variability 10, }\\ & \text{intraspecies variability 10)}=0.45\;\text{ppm} \end{array}$$

Based on the results of the genotoxicity studies, the mechanism of carcinogenicity of EB in animals is considered non-genotoxic. According to DFG MAK [25], an increase in tumor incidence in rats and mice exposed to high EB concentrations resulted in chronic damage to organ functions because EB caused not only increased cell proliferation but also enzyme induction in the target organ. Regarding renal carcinogenicity in rats, the results of histopathological examination in the study by the NTP [19] were reevaluated by two different researchers, according to ATSDR [21]. Hard [77] concluded that the increased incidence of renal cell tumors at the high dose was related to the chemically induced chronic progressive nephropathy, with a minor contributing factor in male rats being α2u-globulin nephropathy. α2u-globulin nephropathy and chronic progressive nephropathy are lesions frequently observed in male and aged rats, respectively, and are rat-specific. Therefore, when the involvement of these rat-specific lesions in the mechanism of renal carcinogenicity was clarified, carcinogenicity would be considered without extrapolation to humans. Nevertheless, Seely et al. [78] concluded that the association between chronic progressive nephropathy and renal tubule cell neoplasms is marginal, and the number of renal tubule cell neoplasms secondary to chronic progressive nephropathy (CPN) would be few. According to ATSDR [21], since 1-phenylethanol, a primary oxidative metabolite of EB, increased the incidence of tumors [renal cell adenomas and adenomas and carcinoma (combined)] only in male rats, the carcinogenic potential of EB was considered to be due to EB itself and/or other reactive oxidative metabolites derived from the 4-ethylphenol pathway. According to AU NICNAS [23], ethylhydroquinone and 4-ethylcatechol, active dihydroxylated EB metabolites, are involved in the mechanism of EB carcinogenesis. According to the DFG MAK [25], metabolism and its relation to toxicity are well described, and it was suggested that the metabolites of the CYP2E1 and CYP2F families were responsible for the induced liver and lung toxicities and that reactive oxygen species (ROS) also played a vital role in the toxicity of EB. Therefore, the mechanism of the carcinogenicity of EB has been discussed in diverse contexts, although it has not yet been definitively determined.

In the derivation of the hazard assessment value under the Chemical Substances Control Law in Japan, an UF of 10 for a severe toxic effect (carcinogenicity with threshold) is applied in addition to the UFs for interspecies and intraspecies variabilities. Nevertheless, the additional UF for carcinogenicity was not applied in the present assessment for the IAQ guideline because we considered that the toxicological severity of carcinogenicity with threshold was equivalent to other toxicities such as general toxicity, and the carcinogenicity potential of EB was considered not so high based on evidence that the incidences of lung tumor in male mice, liver tumors in females mice, and interstitial cell tumor in rat testes were almost similar to historical control data, and renal cell adenoma in female rats was induced only at the highest dose tested. This approach will be standard in the detailed hazard assessment for IAQ guidelines at the CIAP.

## Conclusions and remarks

Since we could not obtain appropriate information on toxicity effects in humans for the quantitative hazard assessment, the hazard assessment values of each toxicity category were derived based on the results of toxicity studies in experimental animals. The NOAEL/LOAEL of each toxicity study was determined, and the most valid key study, endpoint, and PODs were selected for deriving the hazard assessment values for each toxicity category.

Among the representative hazard assessment values of each toxicity category, a minimum value of 0.0858 ppm (370 µg/m^3^: conversion value at 25 °C) for general toxicity based on the loss of OHCs in the organ of Corti in the cochlea was selected as the representative hazard assessment value for EB and suggested as the basis of the revised IAQ guideline value. As described previously, this concentration might prevent adverse auditory effects in humans observed in the epidemiological study [48] and other effects, including developmental toxicity and EB carcinogenicity.

EB has been frequently detected in the past survey of volatile organic compounds in indoor air in Japan, as shown in the Background section [8–10]. A comparison of these survey results with the representative hazard assessment value of 370 µg/m^3^ in the present evaluation suggested that the measured concentrations in several sampling locations did not exceed the hazard assessment value. Nonetheless, the maximum concentrations in some sampling locations exceeded the hazard assessment value. Therefore, we considered that it would be necessary to conduct continuous surveys of indoor air concentrations of EB.

This value for EB was proposed to the MHLW and confirmed as a hazard assessment value at the CIAP in February 2024. Furthermore, the validity of the value was discussed at the CIAP, referring to the results of the indoor air concentration survey in Japan conducted in 2023 (unpublished data). Finally, the value was adopted as a revised IAQ guideline for EB and the revised value was officially notified by the MHLW in January 2025 [79]. Therefore, measures will be taken by stakeholders to ensure that the indoor air concentrations of EB emitted from building materials would not exceed this value.

## Abbreviations

ACGIH: American Conference of Governmental Industrial Hygienists
ANSES: French Agency for Food, Environmental and Occupational Health & Safety
ATSDR: Agency for Toxic Substances and Disease Registry
AU NICNAS: Australian National Industrial Chemicals Notification and Assessment Scheme
CHO: Chinese hamster ovary
CIAP: Committee on Indoor Air Pollution
CPN: chronic progressive nephropathy
DART: Developmental and reproductive toxicity
DFG: Deutsche Forschungsgemeinschaft (German Research Foundation)
EB: ethylbenzene
FOB: functional examination
GHS: Globally Harmonized System of Classification and Labeling of Chemicals
IAQ: indoor air quality
IARC: International Agency for Research on Cancer
IRIS: Integrated Risk Information System
JSOH: Japan Society for Occupational Health
LOAEL: lowest observed adverse effect level
MHLW: the Ministry of Health, Labour and Welfare of the Japanese government
NOAEL: no-observed adverse effect level
OECD: Organization for Economic Co-operation and Development
OHC: outer hair cell
POD: Point of departure
ROS: reactive oxygen species
SHE: Syrian hamster embryo
UF: Uncertain factor
US EPA: United States Environmental Protection Agency
WHO: World Health Organization

## Declarations

### Availability of data and material

Data derived from a source in the public domain (with a link).
